# Metagenome-Assembled Genome Sequence of *Rhodobacteraceae* Bacterium Strain Clear-D3, Assembled from a *Dolichospermum* Consortium from Clear Lake, California

**DOI:** 10.1128/MRA.01119-20

**Published:** 2020-12-03

**Authors:** Chuankai Cheng, Kyra M. Florea, Eric A. Webb, J. Cameron Thrash

**Affiliations:** a Department of Biological Sciences, University of Southern California, Los Angeles, California, USA; Georgia Institute of Technology

## Abstract

Here, we report the metagenome-assembled genome sequence of a *Rhodobacteraceae* bacterium strain, Clear-D3, that was reconstructed from a cyanobacterial enrichment from a eutrophic lake. The draft genome sequence shows evidence of an anoxygenic photoautotrophic lifestyle. Other potential capabilities include aerobic heterotrophy, flagellar motility, chemotaxis, and utilization of complex C-P compounds.

## ANNOUNCEMENT

Cyanobacterial harmful algal blooms (cyanoHABs) pose an environmental threat to freshwater bodies globally. *Dolichospermum* is a common genus of cyanoHAB-forming cyanobacteria. The frequency of *Dolichospermum* blooms has increased in recent years, prompting a need to investigate and understand the biological underpinnings of bloom formation (1). We generated enrichments from *Dolichospermum* aggregates to investigate microbial interactions supporting these common harmful algal bloom (HAB) communities. Metagenomic sequencing and assembly yielded a novel *Rhodobacteraceae* metagenome-assembled genome (MAG) that will help downstream efforts to understand *Dolichospermum* symbioses.

We collected *Dolichospermum* aggregates from surface water using a bucket tow in Clear Lake, CA (lat 38.973166, long 122.72809), in August 2019. Colonies were hand separated, enriched for a total of 7 months in 50% BG-11_0_ medium (50% BG-11_0_ medium contains 1 part BG-11_0_ medium and 1 part sterile Milli-Q water), and incubated at 25°C at 100 μmol Q/m^2^/s on a 12:12-h light/dark cycle, with NaNO_3_ excluded to promote the growth of diazotrophic taxa and their symbionts. Additional medium was added to the enrichments every 2 weeks to maintain growth. Prior to sequencing, we identified the core strain of the aggregates morphologically as a *Dolichospermum* sp. (2) with an Axiostar epifluorescence microscope (Zeiss, Oberkochen, Germany). We extracted DNA from a single enrichment following an adapted Qiagen DNeasy PowerBiofilm kit protocol, modified from the manufacturer’s instructions as follows: after the addition of solution C1, samples were subjected to freeze-thaw (liquid nitrogen [–196°C] to 75°C) four times and then incubated overnight in a proteinase K solution (25 μl of a 20-mg/ml solution). Isolated DNA was verified with Tris-borate-EDTA (TBE) gel electrophoresis and quantified with NanoDrop UV-visible (UV-Vis) spectroscopy and Qubit spectrofluorometry (Thermo Fisher Scientific, Waltham, MA). Illumina library preparation and paired-end (PE) 150-bp sequencing (1 Gbp) were performed with a NEBNext DNA library preparation kit according to the manufacturer's recommendations by Novogene (Nanjing, China) with 300-bp inserts, producing 19,844,532 reads. We processed raw reads through the narrative for genome extraction from shotgun metagenome sequence data on the KBase server (3). Default settings were used for all software unless otherwise noted. We used FastQC v0.11.5 (4) to check the quality of paired-end reads, trimmed sequences with Trimmomatic v0.36 (5) to remove reads under 36 bp, assembled reads with metaSPAdes v3.13.0 (6), and binned the assembled contigs with MaxBin v2.2.4 (7). We annotated Clear-D3 through PGAP (8) and used GTDB-tk v1.1.0 (9) for taxonomic identification with “classify_wf” and the release 95 database. The metabolic potential was analyzed using MetaSanity v1.2 (10) and KEGG Decoder v1.1 (11).

The genome assembly pipeline yielded a 4,519,349-bp MAG we designated Clear-D3, which was taxonomically placed within the provisional genus TH137 in the family *Rhodobacteraceae*. Clear-D3 included 93 contigs (*N*_50_ value, 168,577 bp) with a GC content of 65.25% and 4,396 coding sequences. The average coverage of the contigs was 41.28×. CheckM v1.0.18 (12) predicted the genome as 98.44% complete with 1.73% contamination. Strain Clear-D3 has predicted complete central carbon metabolism (glycolysis, the tricarboxylic acid cycle, and NADH-quinone oxidoreductase), showing its potential for a heterotrophic lifestyle. However, it also has genes for the Calvin-Benson-Bassham cycle, anoxygenic type II reaction centers, and sulfite dehydrogenase (quinone), suggesting the potential for photolithoautotrophy. Furthermore, this MAG shows potential in both transporting (*phnCED*) and utilizing (C-P lyase tetradimer complex *phnJGHI*) phosphonate compounds.

### Data availability.

This whole-genome shotgun project has been deposited at DDBJ/ENA/GenBank under the accession number JACVZW000000000. The version described in this paper is version JACVZW010000000. The BioProject number is PRJNA657201, and the reads are available at the SRA under accession number SRX8961729.
